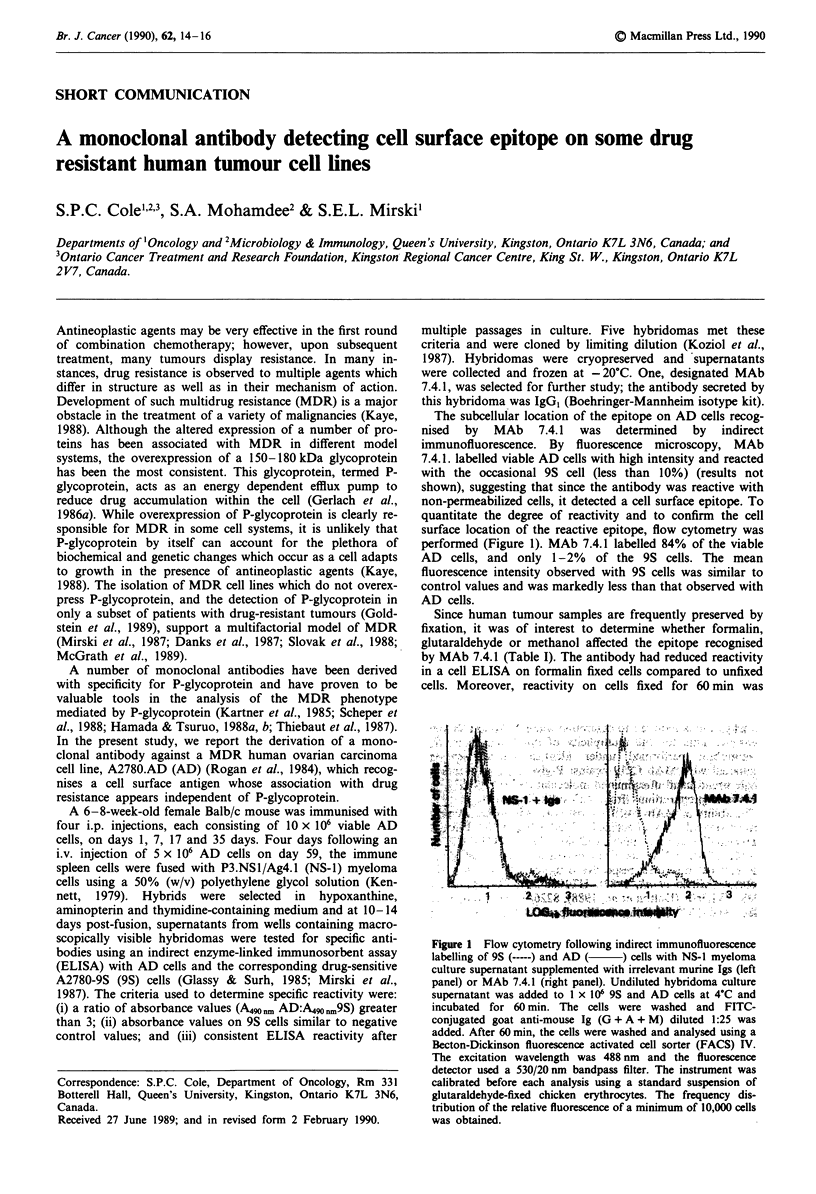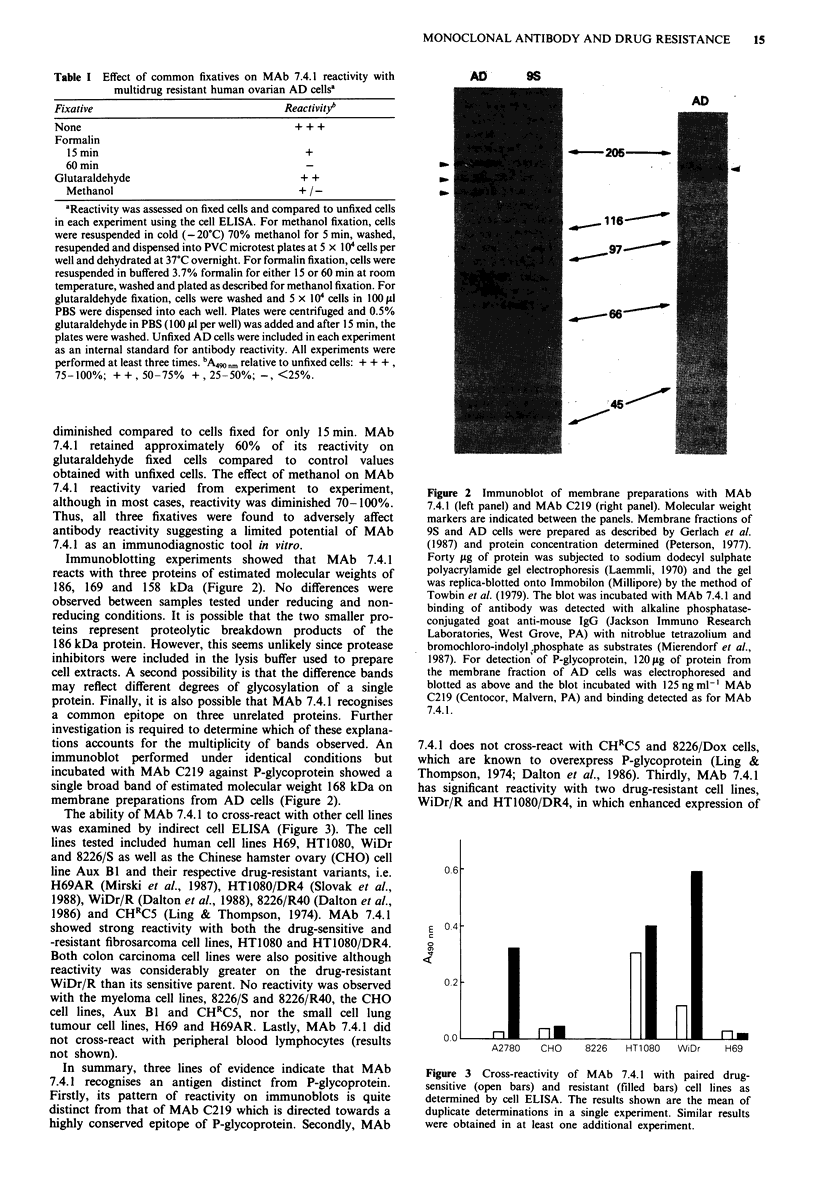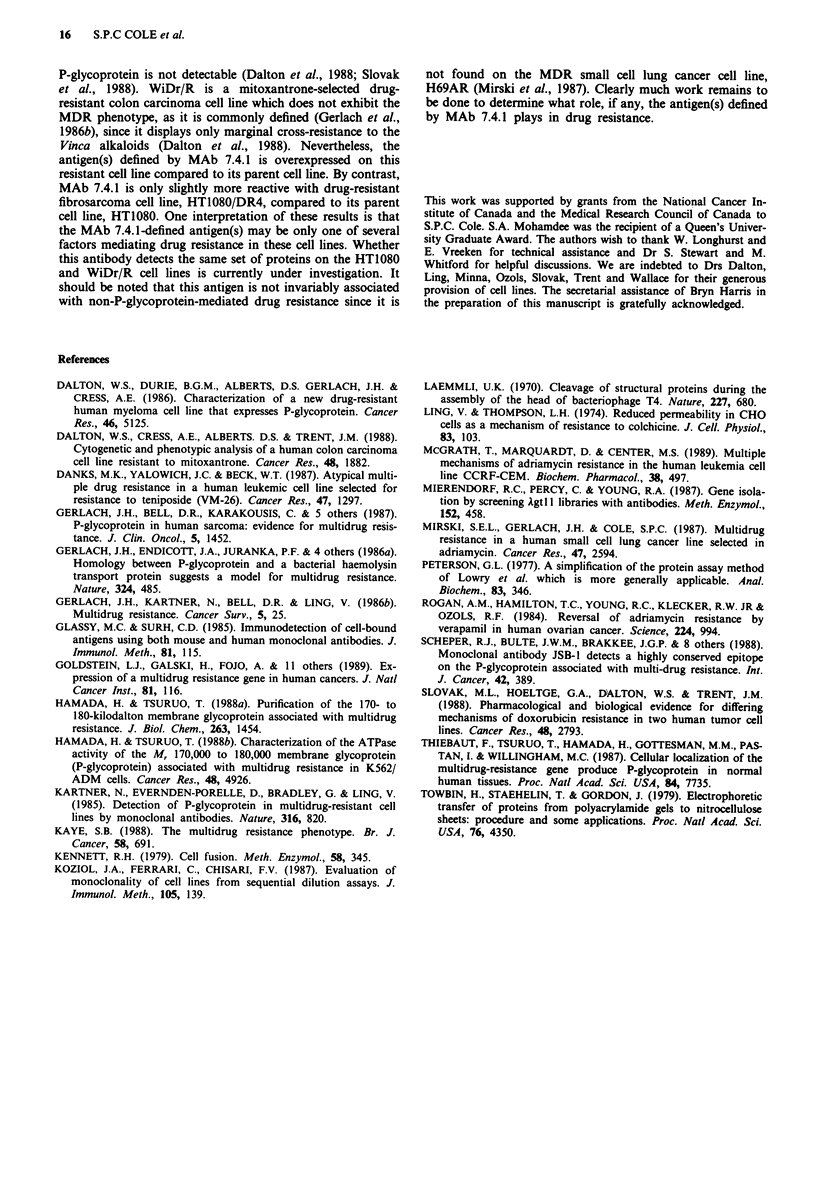# A monoclonal antibody detecting cell surface epitope on some drug resistant human tumour cell lines.

**DOI:** 10.1038/bjc.1990.220

**Published:** 1990-07

**Authors:** S. P. Cole, S. A. Mohamdee, S. E. Mirski

**Affiliations:** Department of Oncology, Queen's University, Kingston, Ontario, Canada.

## Abstract

**Images:**


Br. J. Cancer (1990), 62, 14-16 © Macmillan Press Ltd., 1990

SHORT COMMUNICATION

### A monoclonal antibody detecting cell surface epitope on some drug resistant human tumour cell lines

S.P.C. Cole[1,2,3], S.A. Mohamdee[2] & S.E.L. Mirski[1]

*Departments of [1]Oncology and [2]Microbiology & Immunology, Queen's University, Kingston, Ontario K7L 3N6, Canada; and [3]Ontario Cancer Treatment and Research Foundation, Kingston Regional Cancer Centre, King St. W., Kingston, Ontario K7L 2V7, Canada.*

Antineoplastic agents may be very effective in the first round of combination chemotherapy; however, upon subsequent treatment, many tumours display resistance. In many instances, drug resistance is observed to multiple agents which differ in structure as well as in their mechanism of action. Development of such multidrug resistance (MDR) is a major obstacle in the treatment of a variety of malignancies (Kaye, 1988). Although the altered expression of a number of proteins has been associated with MDR in different model systems, the overexpression of a 150-180 kDa glycoprotein has been the most consistent. This glycoprotein, termed P-glycoprotein, acts as an energy dependent efflux pump to reduce drug accumulation within the cell (Gerlach *et al.*, 1986*a*). While overexpression of P-glycoprotein is clearly responsible for MDR in some cell systems, it is unlikely that P-glycoprotein by itself can account for the plethora of biochemical and genetic changes which occur as a cell adapts to growth in the presence of antineoplastic agents (Kaye, 1988). The isolation of MDR cell lines which do not overexpress P-glycoprotein, and the detection of P-glycoprotein in only a subset of patients with drug-resistant tumours (Goldstein *et al.*, 1989), support a multifactorial model of MDR (Mirski *et al.*, 1987; Danks *et al.*, 1987; Slovak *et al.*, 1988; McGrath *et al.*, 1989).

A number of monoclonal antibodies have been derived with specificity for P-glycoprotein and have proven to be valuable tools in the analysis of the MDR phenotype mediated by P-glycoprotein (Kartner *et al.*, 1985; Scheper *et al.*, 1988; Hamada & Tsuruo, 1988*a*, *b*; Thiebaut *et al.*, 1987). In the present study, we report the derivation of a monoclonal antibody against a MDR human ovarian carcinoma cell line, A2780.AD (AD) (Rogan *et al.*, 1984), which recognises a cell surface antigen whose association with drug resistance appears independent of P-glycoprotein.

A 6-8-week-old female Balb/c mouse was immunised with four i.p. injections, each consisting of $10 \times 10^6$ viable AD cells, on days 1, 7, 17 and 35 days. Four days following an i.v. injection of $5 \times 10^6$ AD cells on day 59, the immune spleen cells were fused with P3.NS1/Ag4.1 (NS-1) myeloma cells using a 50% (w/v) polyethylene glycol solution (Kennett, 1979). Hybrids were selected in hypoxanthine, aminopterin and thymidine-containing medium and at 10-14 days post-fusion, supernatants from wells containing macroscopically visible hybridomas were tested for specific antibodies using an indirect enzyme-linked immunosorbent assay (ELISA) with AD cells and the corresponding drug-sensitive A2780-9S (9S) cells (Glassy & Surh, 1985; Mirski *et al.*, 1987). The criteria used to determine specific reactivity were: (i) a ratio of absorbance values ($A_{490\,nm}$ AD:$A_{490\,nm}$9S) greater than 3; (ii) absorbance values on 9S cells similar to negative control values; and (iii) consistent ELISA reactivity after multiple passages in culture. Five hybridomas met these criteria and were cloned by limiting dilution (Koziol *et al.*, 1987). Hybridomas were cryopreserved and supernatants were collected and frozen at $-20°C$. One, designated MAb 7.4.1, was selected for further study; the antibody secreted by this hybridoma was $IgG_1$ (Boehringer-Mannheim isotype kit).

The subcellular location of the epitope on AD cells recognised by MAb 7.4.1 was determined by indirect immunofluorescence. By fluorescence microscopy, MAb 7.4.1. labelled viable AD cells with high intensity and reacted with the occasional 9S cell (less than 10%) (results not shown), suggesting that since the antibody was reactive with non-permeabilized cells, it detected a cell surface epitope. To quantitate the degree of reactivity and to confirm the cell surface location of the reactive epitope, flow cytometry was performed (Figure 1). MAb 7.4.1 labelled 84% of the viable AD cells, and only 1-2% of the 9S cells. The mean fluorescence intensity observed with 9S cells was similar to control values and was markedly less than that observed with AD cells.

Since human tumour samples are frequently preserved by fixation, it was of interest to determine whether formalin, glutaraldehyde or methanol affected the epitope recognised by MAb 7.4.1 (Table I). The antibody had reduced reactivity in a cell ELISA on formalin fixed cells compared to unfixed cells. Moreover, reactivity on cells fixed for 60 min was

**Figure 1** Flow cytometry following indirect immunofluorescence labelling of 9S (-----) and AD (———) cells with NS-1 myeloma culture supernatant supplemented with irrelevant murine Igs (left panel) or MAb 7.4.1 (right panel). Undiluted hybridoma culture supernatant was added to $1 \times 10^6$ 9S and AD cells at 4°C and incubated for 60 min. The cells were washed and FITC-conjugated goat anti-mouse Ig (G + A + M) diluted 1:25 was added. After 60 min, the cells were washed and analysed using a Becton-Dickinson fluorescence activated cell sorter (FACS) IV. The excitation wavelength was 488 nm and the fluorescence detector used a 530/20 nm bandpass filter. The instrument was calibrated before each analysis using a standard suspension of glutaraldehyde-fixed chicken erythrocytes. The frequency distribution of the relative fluorescence of a minimum of 10,000 cells was obtained.

Correspondence: S.P.C. Cole, Department of Oncology, Rm 331 Botterell Hall, Queen's University, Kingston, Ontario K7L 3N6, Canada.
Received 27 June 1989; and in revised form 2 February 1990.

**Table I** Effect of common fixatives on MAb 7.4.1 reactivity with multidrug resistant human ovarian AD cells[a]

| *Fixative* | *Reactivity*[b] |
|---|---|
| None | + + + |
| Formalin | |
| 15 min | + |
| 60 min | − |
| Glutaraldehyde | + + |
| Methanol | + / − |

[a]Reactivity was assessed on fixed cells and compared to unfixed cells in each experiment using the cell ELISA. For methanol fixation, cells were resuspended in cold (−20°C) 70% methanol for 5 min, washed, resuspended and dispensed into PVC microtest plates at $5 \times 10^4$ cells per well and dehydrated at 37°C overnight. For formalin fixation, cells were resuspended in buffered 3.7% formalin for either 15 or 60 min at room temperature, washed and plated as described for methanol fixation. For glutaraldehyde fixation, cells were washed and $5 \times 10^4$ cells in 100 µl PBS were dispensed into each well. Plates were centrifuged and 0.5% glutaraldehyde in PBS (100 µl per well) was added and after 15 min, the plates were washed. Unfixed AD cells were included in each experiment as an internal standard for antibody reactivity. All experiments were performed at least three times. [b]$A_{490\,nm}$ relative to unfixed cells: + + +, 75–100%; + +, 50–75% +, 25–50%; −, <25%.

diminished compared to cells fixed for only 15 min. MAb 7.4.1 retained approximately 60% of its reactivity on glutaraldehyde fixed cells compared to control values obtained with unfixed cells. The effect of methanol on MAb 7.4.1 reactivity varied from experiment to experiment, although in most cases, reactivity was diminished 70–100%. Thus, all three fixatives were found to adversely affect antibody reactivity suggesting a limited potential of MAb 7.4.1 as an immunodiagnostic tool *in vitro*.

Immunoblotting experiments showed that MAb 7.4.1 reacts with three proteins of estimated molecular weights of 186, 169 and 158 kDa (Figure 2). No differences were observed between samples tested under reducing and non-reducing conditions. It is possible that the two smaller proteins represent proteolytic breakdown products of the 186 kDa protein. However, this seems unlikely since protease inhibitors were included in the lysis buffer used to prepare cell extracts. A second possibility is that the difference bands may reflect different degrees of glycosylation of a single protein. Finally, it is also possible that MAb 7.4.1 recognises a common epitope on three unrelated proteins. Further investigation is required to determine which of these explanations accounts for the multiplicity of bands observed. An immunoblot performed under identical conditions but incubated with MAb C219 against P-glycoprotein showed a single broad band of estimated molecular weight 168 kDa on membrane preparations from AD cells (Figure 2).

The ability of MAb 7.4.1 to cross-react with other cell lines was examined by indirect cell ELISA (Figure 3). The cell lines tested included human cell lines H69, HT1080, WiDr and 8226/S as well as the Chinese hamster ovary (CHO) cell line Aux B1 and their respective drug-resistant variants, i.e. H69AR (Mirski *et al.*, 1987), HT1080/DR4 (Slovak *et al.*, 1988), WiDr/R (Dalton *et al.*, 1988), 8226/R40 (Dalton *et al.*, 1986) and CH[R]C5 (Ling & Thompson, 1974). MAb 7.4.1 showed strong reactivity with both the drug-sensitive and -resistant fibrosarcoma cell lines, HT1080 and HT1080/DR4. Both colon carcinoma cell lines were also positive although reactivity was considerably greater on the drug-resistant WiDr/R than its sensitive parent. No reactivity was observed with the myeloma cell lines, 8226/S and 8226/R40, the CHO cell lines, Aux B1 and CH[R]C5, nor the small cell lung tumour cell lines, H69 and H69AR. Lastly, MAb 7.4.1 did not cross-react with peripheral blood lymphocytes (results not shown).

In summary, three lines of evidence indicate that MAb 7.4.1 recognises an antigen distinct from P-glycoprotein. Firstly, its pattern of reactivity on immunoblots is quite distinct from that of MAb C219 which is directed towards a highly conserved epitope of P-glycoprotein. Secondly, MAb

**Figure 2** Immunoblot of membrane preparations with MAb 7.4.1 (left panel) and MAb C219 (right panel). Molecular weight markers are indicated between the panels. Membrane fractions of 9S and AD cells were prepared as described by Gerlach *et al.* (1987) and protein concentration determined (Peterson, 1977). Forty µg of protein was subjected to sodium dodecyl sulphate polyacrylamide gel electrophoresis (Laemmli, 1970) and the gel was replica-blotted onto Immobilon (Millipore) by the method of Towbin *et al.* (1979). The blot was incubated with MAb 7.4.1 and binding of antibody was detected with alkaline phosphatase-conjugated goat anti-mouse IgG (Jackson Immuno Research Laboratories, West Grove, PA) with nitroblue tetrazolium and bromochloro-indolyl phosphate as substrates (Mierendorf *et al.*, 1987). For detection of P-glycoprotein, 120 µg of protein from the membrane fraction of AD cells was electrophoresed and blotted as above and the blot incubated with 125 ng ml$^{-1}$ MAb C219 (Centocor, Malvern, PA) and binding detected as for MAb 7.4.1.

7.4.1 does not cross-react with CH[R]C5 and 8226/Dox cells, which are known to overexpress P-glycoprotein (Ling & Thompson, 1974; Dalton *et al.*, 1986). Thirdly, MAb 7.4.1 has significant reactivity with two drug-resistant cell lines, WiDr/R and HT1080/DR4, in which enhanced expression of

**Figure 3** Cross-reactivity of MAb 7.4.1 with paired drug-sensitive (open bars) and resistant (filled bars) cell lines as determined by cell ELISA. The results shown are the mean of duplicate determinations in a single experiment. Similar results were obtained in at least one additional experiment.

P-glycoprotein is not detectable (Dalton *et al.*, 1988; Slovak *et al.*, 1988). WiDr/R is a mitoxantrone-selected drug-resistant colon carcinoma cell line which does not exhibit the MDR phenotype, as it is commonly defined (Gerlach *et al.*, 1986*b*), since it displays only marginal cross-resistance to the *Vinca* alkaloids (Dalton *et al.*, 1988). Nevertheless, the antigen(s) defined by MAb 7.4.1 is overexpressed on this resistant cell line compared to its parent cell line. By contrast, MAb 7.4.1 is only slightly more reactive with drug-resistant fibrosarcoma cell line, HT1080/DR4, compared to its parent cell line, HT1080. One interpretation of these results is that the MAb 7.4.1-defined antigen(s) may be only one of several factors mediating drug resistance in these cell lines. Whether this antibody detects the same set of proteins on the HT1080 and WiDr/R cell lines is currently under investigation. It should be noted that this antigen is not invariably associated with non-P-glycoprotein-mediated drug resistance since it is not found on the MDR small cell lung cancer cell line, H69AR (Mirski *et al.*, 1987). Clearly much work remains to be done to determine what role, if any, the antigen(s) defined by MAb 7.4.1 plays in drug resistance.

This work was supported by grants from the National Cancer Institute of Canada and the Medical Research Council of Canada to S.P.C. Cole. S.A. Mohamdee was the recipient of a Queen's University Graduate Award. The authors wish to thank W. Longhurst and E. Vreeken for technical assistance and Dr S. Stewart and M. Whitford for helpful discussions. We are indebted to Drs Dalton, Ling, Minna, Ozols, Slovak, Trent and Wallace for their generous provision of cell lines. The secretarial assistance of Bryn Harris in the preparation of this manuscript is gratefully acknowledged.

### References

DALTON, W.S., DURIE, B.G.M., ALBERTS, D.S. GERLACH, J.H. & CRESS, A.E. (1986). Characterization of a new drug-resistant human myeloma cell line that expresses P-glycoprotein. *Cancer Res.*, **46**, 5125.

DALTON, W.S., CRESS, A.E., ALBERTS. D.S. & TRENT, J.M. (1988). Cytogenetic and phenotypic analysis of a human colon carcinoma cell line resistant to mitoxantrone. *Cancer Res.*, **48**, 1882.

DANKS, M.K., YALOWICH, J.C. & BECK, W.T. (1987). Atypical multiple drug resistance in a human leukemic cell line selected for resistance to teniposide (VM-26). *Cancer Res.*, **47**, 1297.

GERLACH, J.H., BELL, D.R., KARAKOUSIS, C. & 5 others (1987). P-glycoprotein in human sarcoma: evidence for multidrug resistance. *J. Clin. Oncol.*, **5**, 1452.

GERLACH, J.H., ENDICOTT, J.A., JURANKA, P.F. & 4 others (1986*a*). Homology between P-glycoprotein and a bacterial haemolysin transport protein suggests a model for multidrug resistance. *Nature*, **324**, 485.

GERLACH, J.H., KARTNER, N., BELL, D.R. & LING, V. (1986*b*). Multidrug resistance. *Cancer Surv.*, **5**, 25.

GLASSY, M.C. & SURH, C.D. (1985). Immunodetection of cell-bound antigens using both mouse and human monoclonal antibodies. *J. Immunol. Meth.*, **81**, 115.

GOLDSTEIN, L.J., GALSKI, H., FOJO, A. & 11 others (1989). Expression of a multidrug resistance gene in human cancers. *J. Natl Cancer Inst.*, **81**, 116.

HAMADA, H. & TSURUO, T. (1988*a*). Purification of the 170- to 180-kilodalton membrane glycoprotein associated with multidrug resistance. *J. Biol. Chem.*, **263**, 1454.

HAMADA, H. & TSURUO, T. (1988*b*). Characterization of the ATPase activity of the $M_r$ 170,000 to 180,000 membrane glycoprotein (P-glycoprotein) associated with multidrug resistance in K562/ADM cells. *Cancer Res.*, **48**, 4926.

KARTNER, N., EVERNDEN-PORELLE, D., BRADLEY, G. & LING, V. (1985). Detection of P-glycoprotein in multidrug-resistant cell lines by monoclonal antibodies. *Nature*, **316**, 820.

KAYE, S.B. (1988). The multidrug resistance phenotype. *Br. J. Cancer*, **58**, 691.

KENNETT, R.H. (1979). Cell fusion. *Meth. Enzymol.*, **58**, 345.

KOZIOL, J.A., FERRARI, C., CHISARI, F.V. (1987). Evaluation of monoclonality of cell lines from sequential dilution assays. *J. Immunol. Meth.*, **105**, 139.

LAEMMLI, U.K. (1970). Cleavage of structural proteins during the assembly of the head of bacteriophage T4. *Nature*, **227**, 680.

LING, V. & THOMPSON, L.H. (1974). Reduced permeability in CHO cells as a mechanism of resistance to colchicine. *J. Cell. Physiol.*, **83**, 103.

MCGRATH, T., MARQUARDT, D. & CENTER, M.S. (1989). Multiple mechanisms of adriamycin resistance in the human leukemia cell line CCRF-CEM. *Biochem. Pharmacol.*, **38**, 497.

MIERENDORF, R.C., PERCY, C. & YOUNG, R.A. (1987). Gene isolation by screening λgt11 libraries with antibodies. *Meth. Enzymol.*, **152**, 458.

MIRSKI, S.E.L., GERLACH, J.H. & COLE, S.P.C. (1987). Multidrug resistance in a human small cell lung cancer line selected in adriamycin. *Cancer Res.*, **47**, 2594.

PETERSON, G.L. (1977). A simplification of the protein assay method of Lowry *et al.* which is more generally applicable. *Anal. Biochem.*, **83**, 346.

ROGAN, A.M., HAMILTON, T.C., YOUNG, R.C., KLECKER, R.W. JR & OZOLS, R.F. (1984). Reversal of adriamycin resistance by verapamil in human ovarian cancer. *Science*, **224**, 994.

SCHEPER, R.J., BULTE, J.W.M., BRAKKEE, J.G.P. & 8 others (1988). Monoclonal antibody JSB-1 detects a highly conserved epitope on the P-glycoprotein associated with multi-drug resistance. *Int. J. Cancer*, **42**, 389.

SLOVAK, M.L., HOELTGE, G.A., DALTON, W.S. & TRENT, J.M. (1988). Pharmacological and biological evidence for differing mechanisms of doxorubicin resistance in two human tumor cell lines. *Cancer Res.*, **48**, 2793.

THIEBAUT, F., TSURUO, T., HAMADA, H., GOTTESMAN, M.M., PASTAN, I. & WILLINGHAM, M.C. (1987). Cellular localization of the multidrug-resistance gene produce P-glycoprotein in normal human tissues. *Proc. Natl Acad. Sci. USA*, **84**, 7735.

TOWBIN, H., STAEHELIN, T. & GORDON, J. (1979). Electrophoretic transfer of proteins from polyacrylamide gels to nitrocellulose sheets: procedure and some applications. *Proc. Natl Acad. Sci. USA*, **76**, 4350.